# An uncertainty and sensitivity analysis approach for GIS-based multicriteria landslide susceptibility mapping

**DOI:** 10.1080/13658816.2013.869821

**Published:** 2014-01-20

**Authors:** Bakhtiar Feizizadeh, Thomas Blaschke

**Affiliations:** ^a^ Department of Geoinformatics – Z_GIS, University of Salzburg, Salzburg, Austria; ^b^ Center for Remote Sensing and GIS, University of Tabriz, Tabriz, Iran

**Keywords:** landslide susceptibility mapping, GIS-MCDA, Monte Carlo simulation, sensitivity analysis, Dempster–Shafer Theory, Urmia lake basin

## Abstract

GIS-based multicriteria decision analysis (MCDA) methods are increasingly being used in landslide susceptibility mapping. However, the uncertainties that are associated with MCDA techniques may significantly impact the results. This may sometimes lead to inaccurate outcomes and undesirable consequences. This article introduces a new GIS-based MCDA approach. We illustrate the consequences of applying different MCDA methods within a decision-making process through uncertainty analysis. Three GIS-MCDA methods in conjunction with Monte Carlo simulation (MCS) and Dempster–Shafer theory are analyzed for landslide susceptibility mapping (LSM) in the Urmia lake basin in Iran, which is highly susceptible to landslide hazards. The methodology comprises three stages. First, the LSM criteria are ranked and a sensitivity analysis is implemented to simulate error propagation based on the MCS. The resulting weights are expressed through probability density functions. Accordingly, within the second stage, three MCDA methods, namely analytical hierarchy process (AHP), weighted linear combination (WLC) and ordered weighted average (OWA), are used to produce the landslide susceptibility maps. In the third stage, accuracy assessments are carried out and the uncertainties of the different results are measured. We compare the accuracies of the three MCDA methods based on (1) the Dempster–Shafer theory and (2) a validation of the results using an inventory of known landslides and their respective coverage based on object-based image analysis of IRS-ID satellite images. The results of this study reveal that through the integration of GIS and MCDA models, it is possible to identify strategies for choosing an appropriate method for LSM. Furthermore, our findings indicate that the integration of MCDA and MCS can significantly improve the accuracy of the results. In LSM, the AHP method performed best, while the OWA reveals better performance in the reliability assessment. The WLC operation yielded poor results.

## 1. Introduction

Multicriteria decision analysis (MCDA) is one of the most fundamental decision support operations in GIS (Jiang and Eastman 2000). The capability of MCDA, when integrated with GIS, makes GIS-based MCDA one of the most useful methods for spatial planning and management (Joerin *et al.* 2001, Chen *et al.* 2007, 2009, Karnatak *et al.* 2007). GIS-MCDA can be defined as ‘a process that transforms and combines geographical data and value judgments (the decision-maker’s preferences) to obtain information for decision-making’ (Malczewski 2006, p. 703). The GIS-MCDA process consists of procedures that involve the utilization of geographical data, as well as the decision-maker’s preferences, and the manipulation of these data and preferences (Farkas 2009). MCDA methods have become important tools in terms of the management sciences and operations research (Drobne and Lisec 2009). MCDA provides a rich collection of procedures and algorithms for structuring decision problems, as well as designing, evaluating, and prioritizing alternative decisions. It is in the context of the synergetic capabilities of GIS and MCDA that one can observe the benefits for advancing theoretical and applied research on the integration of GIS and MCDA (Malczewski 1999, 2006, Boroushaki and Malczewski 2010).

It is increasingly recognized that MCDA’s outcomes are prone to the inherent uncertainties related to the MCDA process (Feizizadeh *et al.* 2012). The principal sources of GIS-MCDA uncertainty are due to errors and variability in model choice, system understanding, weighting factors, data used, and human judgment (Crosetto *et al.* 2000). Malczewski (2006) noted that many real-world decisions are uncertain because they involve some aspects with unknown uncertainties in decision-making (Comber *et al.* 2010). He identified two basic types of uncertainty that may be associated with a decision situation including ‘(1) uncertainty associated with limited information about the decision situation and (2) uncertainty associated with fuzziness (imprecision) concerning the description of the semantic meaning of the events, phenomena, or statements themselves’ (Malczewski 2006, p. 713). Even though uncertainty in GIS-MCDA may come from various sources (Malczewski 2006), we need to assume a high influence of the criteria weighting process. Criteria weights are often the greatest contributor to controversy and uncertainty. This could be because decision-makers are not totally aware of their preferences regarding the criteria or perhaps because nature and scale of the criteria is not known. Furthermore, especially when multiple decision-makers are involved, it is often not possible to derive only one set of weights, but rather ranges of weights, thus producing more than one set of results (Chen and Zhu 2010). Even small changes in decision weights and methods may have a signiﬁcant impact on the rank ordering of the criteria and accordingly the results of the GIS-MCDA, which sometimes leads to inaccurate outcomes and undesirable consequences (Feizizadeh and Blaschke 2013a). To reduce the chance of error in GIS-MCDA methods, uncertainty analysis is a process that leads to the assessment of the reliability of MCDA’s results in both quantitative and qualitative approaches. Within this study, we therefore aim to contribute to a better understanding of the uncertainties inherent to GIS-MCDA methods and to increase the stability of their outputs by illustrating the impact of small changes to speciﬁc input parameters on evaluation outcomes.

In this article, we start from the hypothesis that GIS-MCDA uncertainty analysis provides a possibility of measuring the level of confidence in the decision-maker (Chen *et al.* 2011). MCDA uncertainty analysis embraces issues beyond traditional risk definitions. These broader issues include the propagation of errors in predictive environmental models (Oberkampf *et al.* 2004, Benke and Pelizaro 2010). Such issues require an analysis of probability distributions, rather than a risk specification based on a single imprecise probability or consequence (Benke *et al.* 2007). One specific approach to uncertainty analysis applicable to MCDA is to gain a sense of error or uncertainty in the predictions, given the uncertainty in the criterion weights (Benke and Pelizaro 2010). It is believed that it is essential to handle GIS-MCDA errors and uncertainty in decision-making, particularly when decisions are based on probabilistic ranges rather than on deterministic results (Aerts *et al.* 2003, Tenerelli and Carver 2012). The combination of uncertainty and sensitivity analyses is crucial to the validation and calibration of MCDA (Chen *et al.* 2009). Uncertainty analysis aims to identify and quantify confidence intervals for a model output by assessing the response to uncertainties in the model inputs (Crosetto *et al.* 2000). Sensitivity analysis has also been defined as a process that aims to assess the response of a model to changes in input parameters (Ligmann-Zielinska and Jankowski 2008). Technically, sensitivity analysis partitions the results, under different conditions of the model components and parameters, for identifying the key determining variables (Smith 2002). It can help to reduce uncertainty in MCDA and to increase the stability of its outputs. This is achieved by illustrating the impact of introducing small changes to specific input parameters on evaluation outcomes (Crosetto *et al.* 2000, Crosetto and Tarantola 2001, Chen *et al.* 2010, Ravalico *et al.* 2010). However, the combination of uncertainty and sensitivity analyses aims for a better understanding of the respective influences of the assumptions and input parameters of a model (Crosetto *et al.* 2000).

In the context of applying GIS-MCDA to landslide susceptibility mapping (LSM), the authors already employed several MCDA methods and analyzed their particular capabilities and limitations (Feizizadeh *et al.* 2013a,  Feizizadeh and Blaschke 2013b). Our earlier studies revealed that GIS-MCDA methods are highly sensitive to the parameter selection and weighting processes in the decision-making stage (see Feizizadeh *et al.* 2012). Building on this earlier works, in the remainder of this article, we carry out a GIS-MCDA study for LSM with emphasis on the respective sensitivity and uncertainty analyses. The aim is to improve the accuracy of the results while identifying and minimizing the uncertainties associated with the respective MCDA methods. We employ three conventional GIS-MCDA methods and compare the results with the respective results of our proposed novel approach, which integrates sensitivity and uncertainty analyses.

## 2. Study area and data

**Figure 1.**
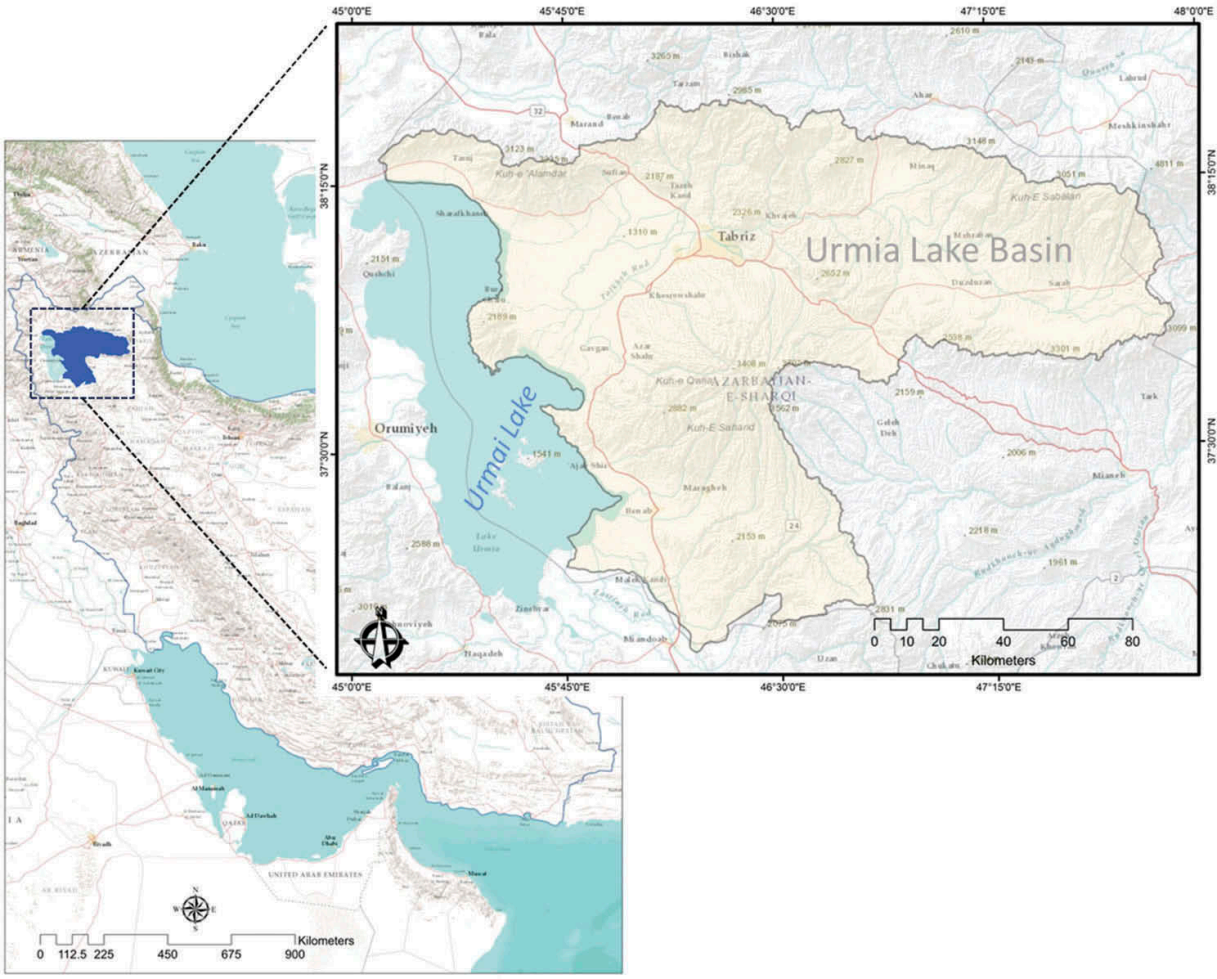
Location of the case study area in Iran (left) and within northern Iran (right).

The study area was the Urmia lake basin, which is located in the north-west of Iran (see Figure 1). The study area with a size of 19,913 km^2^ and 3.2 million inhabitants is important for the East Azerbaijan province in terms of housing, as well as industrial and agricultural activities. The elevation increases from 1260 m at Urmia Lake to 3710 m above sea level in the Sahand Mountains. The climate of this area is semi-arid and the annual precipitation is about 300 mm (Feizizadeh and Blaschke 2013a). Landslides are common in the Urmia lake basin, and the complexity of the geological structure in the associated lithological units, comprised of several formations, causes volcanic hazards, earthquakes, and landslides (Feizizadeh and Blaschke 2011). A landslide inventory database for the East Azerbaijan Province lists 132 known landslide events (MNR *et al.* 2010). The geophysical setting makes the slopes of this area potentially vulnerable to mass movements such as rock fall, creeps, flows, topples, and landslides. In this study, topography, geology, climate, vegetation, and anthropogenic factors were selected based on field studies related to active landslides. Based on this fieldwork and on expert knowledge, geological lineaments and artifacts such as roads were included. The selection of the nine causal factors finally used in this study is based on these four main criteria (topography, geology, climate, vegetation, and anthropogenic factors). A more detailed description of the physical properties of the study area and of the criteria selection strategy is presented in Feizizadeh *et al.* (2013a)  and Feizizadeh and Blaschke (2013a). After all necessary geometric and thematic editing, topology correction and simple basic calculations (e.g., buffers around faults, streams, and roads) were completed, all datasets were arranged in ArcGIS software as raster maps with a resolution of 20 m for further analysis.

## 3. Methodology

### 3.1. GIS-MCDA and uncertainty analysis: workflow

The integration of MCDA techniques with GIS has considerably advanced the conventional map overlay approaches (Malczewski 2004). Over the last 20 years or so, there have been a number of multi-criteria decision rules implemented in the GIS environment, including the weighted linear combination (WLC) or weighted summation/Boolean overlay methods (e.g., Carver 1991), the analytical hierarchy process (AHP) (e.g., Saaty 1977), the ideal/reference point method (e.g., Joerin *et al.* 2001), and the ordered weighted average (OWA) method (Boroushaki and Malczewski 2010, see Malczewski 2006 for an overview of the GIS-MCDA methods). However, there are many ways in which decision criteria can be combined in MCDA. WLC and its variants (Carver 1991, Eastman 1997, 2006, Drobne and Lisec 2009) and OWA require summation of the weighted criteria. The AHP is considered to be the primary MCDA method. In combination with WLC and OWA, AHP can be used in two distinctive ways within a GIS environment: first, it can be employed to derive the weights associated with criteria map layers and, secondly, the AHP principle can be used to aggregate the priority for a particular hierarchical level as well as for alternative levels (Saaty 1977, Banai 1993). Concordance–discordance analyses are methods in which each pair of alternatives, represented as raster pixels or polygons, is analyzed to determine the degree to which one outranks the other in the specified criteria (Drobne and Lisec 2009).

Combining GIS and MCDA is a powerful approach to LSM, and GIS-MCDA methods are increasingly being used in LSM for the prediction of future hazards, decision-making, and hazard mitigation plans (Kritikos and Davies 2011, Pourghasemi *et al.* 2012a, Feizizadeh *et al*. 2013b, Feizizadeh and Blaschke 2013a). Table 1 lists categories of MCDA methods based on their combination rules. Starting from Malczewski (2006), the most relevant literature was analyzed in regard to GIS-MCDA decision rules (e.g., Boolean and Fuzzy) for LSM. In addition, as mentioned in the introduction section, uncertainty is inevitable in MCDA, which emphasizes the importance of assessing the associated uncertainty of each method. However, these approaches have rarely been studied for MCDA methods, particularly for LSM. In an effort to investigate the inherent uncertainties in GIS-MCDA, the Monte Carlo simulation (MCS) and Dempster–Shafer theory (DST) are used to measure the uncertainty both spatially and quantitatively, aiming for a flexible representation of uncertainties for data sources of different types and, in particular, of the underlying expert judgments. Multiple experts’ knowledge is considered to provide more reliable information for an observation (e.g., the failure probability of a component) than a single expert’s knowledge. Nevertheless, expert judgments can suffer from incompleteness and conflicts. DST addresses these issues effectively and is able to combine multi-expert knowledge by taking into account ignorance and conflicts through a belief structure (Sallak *et al.* 2010).

**Table 1.** Classification of GIS-MCDA based on their combination rules and applied methods for LSM.

	MCDA-Combination rules	Type of MCDA	Examples in the literature for LSM
MADA	Weighted summation/Boolean overlay	WLC	Feizizadeh and Blaschke (2013a), Kritikos and Davies (2011)
	Ideal/reference point	TOPSIS, MOLA	
	Analytical hierarchy process	AHP, Fuzzy-AHP	Feizizadeh *et al.* (2012a, 2014), Pourghasemi *et al.* (2012b), Gorsevski and Jankowski (2010), Oh and Pradhan (2011), Kritikos and Davies (2011)
	Outranking methods	ELECTRE, PROMETHEE	
MODA	Multi-objectives programming algorithms (linear-integer programming)		
	Heuristic/search/evolutionary/genetic algorithms		
	Goal programming/reference point algorithms		
Ordered Weighted Average			Feizizadeh *et al.* (2012b, 2014), Feizizadeh and Blaschke (2013a), Kritikos and Davies (2011)
Dempster–Shafer Theory, weights-of-evidence			Gorsevski and Jankowski (2005), Park (2011), Feizizadeh *et al.* (2012b), 2014, Althuwaynee *et al.* (2012), Pourghasemi *et al.* (2012b)
Artificial neural networks, Support vector machines			Yilmaz (2010)
Fuzzy set- MCDA and Kalman filter			Gorsevski and Jankowski (2010), Oh and Pradhan (2011)
Logistic regression			Ayalew and Yamagishi (2005)

Notes: MADA, Multi-attribute decision analysis; MODA, multi-objective decision analysis.

This table is adapted based on MCDA classification in the references of Malczewski (2006).

In this study, we want to analyze the described uncertainties for three different LSM methods (AHP, WLC, and OWA). The methodology is composed of three stages as following:

- The first stage ranks the LSM criteria and sensitivity analyses based on the AHP technique to simulate the error propagation. We employ an AHP pairwise matrix to derive the criteria weights. Then, the MCS assesses the sensitivity weight space, whereby weights are expressed through probability density functions (PDFs).
- Within the second stage, the three MCDA methods are used to produce the LSM in order to optimize and quantitatively and qualitatively assess the results. In the second approaches, we pursue two approaches. The first (‘conventional’) approach is based on the common MCDA, which assesses the susceptibility of different areas to potential landslide hazards by considering nine causal criteria. This approach allows multiple and often conflicting criteria to be taken into account and weights to be applied to input criteria depending on the level of importance ascribed to these by the user (Carver 1991, Tenerelli and Carver 2012). In the second (‘novel’) approach, we aim to produce the landslide susceptibility maps based on the results of the sensitivity analyses and the outcomes of MCS.
- In the third stage, the accuracy of the respective methods are assessed by (1) applying DST and (2) through a validation of the results using the landslide inventory database. The accuracies of the AHP, WLC, and OWA methods for LSM are investigated in respect to the improved respective accuracies, based on the sensitivity and uncertainty analyses.

**Figure 2.**
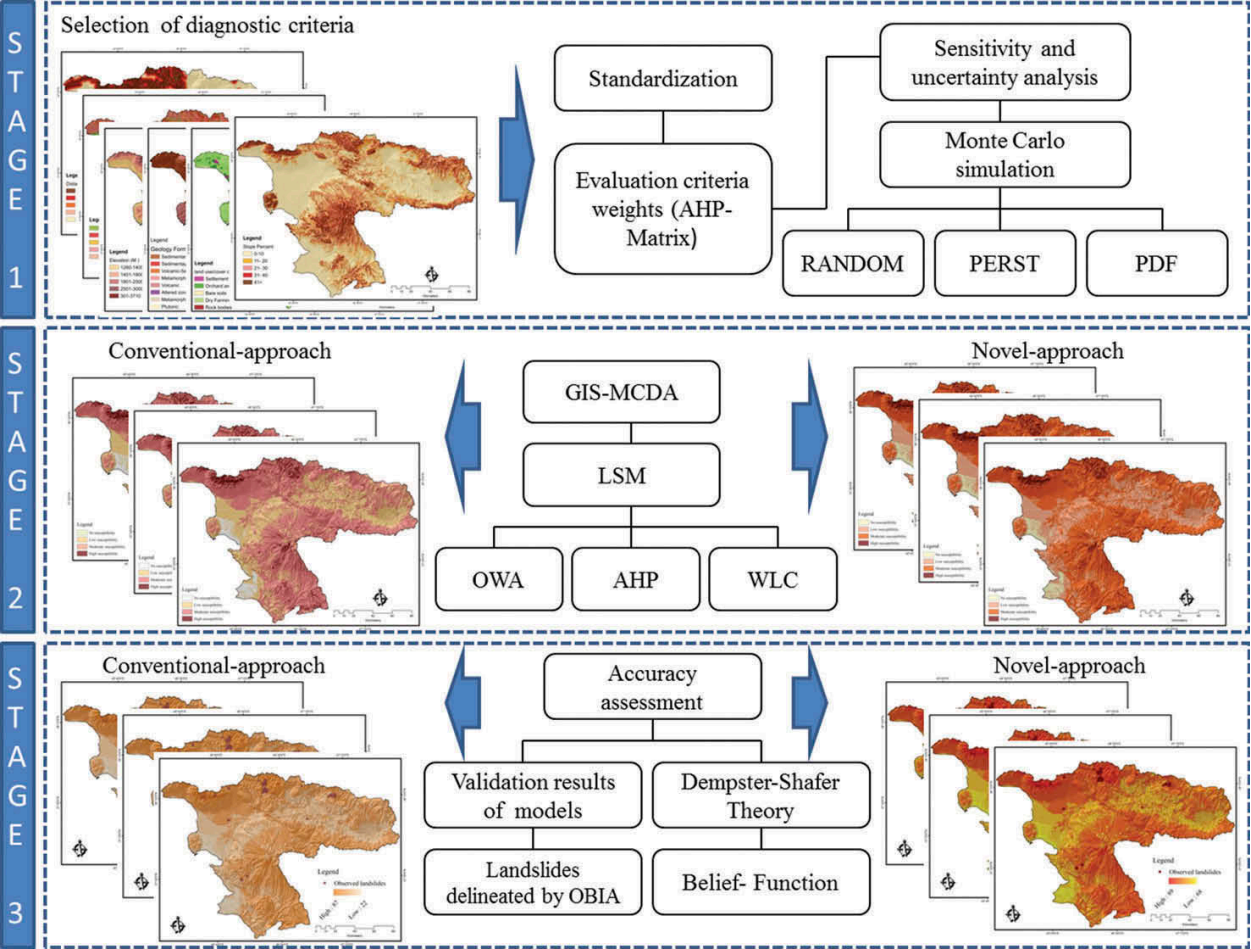
Methodology flowchart of the research.

Figure 2 summarizes the main steps that are used as the methodology of this research.

### 3.2. Criteria standardization

In our LSM decision model, each criterion is represented by a raster map. This includes classified maps based on categorical data (e.g., land use or geology) and value maps (e.g., slope or elevation). Hence, the values and classes of all maps are to be converted into a common scale to reduce the dimensionality. Such conversion is called standardization (Azizur Rahman *et al.* 2012). Within this approach, the criteria are standardized before combined according to the nature of the pairwise comparison technique, which is typically used for rating and standardizing the ordinal values (Malczewski 2004). The first step involves standardizing the predictor variables to a common numeric range using fuzzy membership functions (Jiang and Eastman 2000). In our research, criteria at the lowest level, and the resulting memberships of different potential classes, were subsequently standardized using the maximum eigenvectors approach on a 0 to 1 scale. A fuzzy set is essentially a set whose members may have degrees of membership between 0 and 1, as opposed to a classic binary set in which each element must have either 0 or 1 as the membership degree (Malczewski 2004). Based on this approach, the cells in a map, which are highly suitable for achieving the goal, obtain high standardized values, and less suitable cells obtain low values (Azizur Rahman *et al.* 2012).

### 3.3. Assessing the weights: obtaining decision rules through AHP

Criteria weights represent the inﬂuence of each criterion in the model on the distribution (Robinson *et al.* 2010). In GIS-MCDA, criteria weights are subjectively deﬁned and can affect decision outcomes substantially. They are often the source of great controversy and uncertainty, especially in pluralistic decision-making contexts (Belton and Hodgkin 1999). Since the weights are reﬂective of the relative importance of each criterion, they were assigned to all criteria. The AHP (Saaty 1977) can be applied to help decision-makers make pairwise comparisons of the criteria and to assign appropriate weights for the individual factors being considered and thus deal with this type of vagueness (Li and Li 2009). The AHP methodology provides a hierarchical mechanism for combining expert opinions in order to derive the standard weights of the criteria (Ghosh *et al.* 2011). In the AHP, the weights for the criteria are calculated separately for each hierarchical level. The AHP allocates a pairwise comparison matrix for each hierarchical level (Kordi and Brandt 2012). Within this process, these comparison matrices are defined by ranking the criteria of the corresponding level over each other. Decision-makers determine the ranking of the criteria on the basis of the importance of each criterion over the other, considering their significance in the problem (Kordi and Brandt 2012). GIS-MCDA with weight assignment using AHP provides the capability to produce a decision support tool that (1) blends or integrates both subjective and objective data, (2) models consensus for groups of subject matter experts or stakeholders, and (3) for decision criteria, accounts for inconsistent or possibly incomplete judgments and preferences in the analysis of the possible alternatives (Benke and Pelizaro 2010).

In the application of the AHP method, it is important that the weights derived from a pairwise comparison matrix are consistent. Therefore, one of the strengths of AHP is that it allows for inconsistent relationships, while, at the same time, providing a consistency ratio (CR) as an indicator for the degree of consistency or inconsistency (Forman and Selly 2001, Chen *et al.* 2010, Feizizadeh and Blaschke 2013b). The CR is used to indicate the likelihood that the matrix judgments were generated randomly (Saaty 1977, Park *et al.* 2011):

(1) 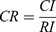

where random index (*RI*) is the consistency index of the randomly generated pairwise comparison matrix according to the number of elements being compared (Drobne and Lisec 2009). Table 2 shows the *RI* for different numbers of criteria given by Saaty (1977). Further, the *CI* can be expressed as

(2) 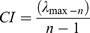

**Table 2.** The mean consistency index of randomly generated matrices (Saaty 1977).

*n*	1	2	3	4	5	6	7	
RI	0	0	0.52	0.89	1.12	1.26	1.36	
*n*	8	9	10	11	12	13	14	15
RI	1.41	1.46	1.49	1.52	1.54	1.56	1.58	1.59

in which *λ*_max_ is the largest or principal eigenvalue of the matrix and *n* is the order of the matrix. A CR of 0.10 or less indicates a reasonable level of consistency (Saaty 1977, Park *et al.* 2011). If the CR < 0.10, it deems that the pairwise comparison matrix has an acceptable consistency and that the weight values are valid and can be utilized. Otherwise, if the *CR* ≥ 0.10, it means that the pairwise comparisons lack consistency and the matrix needs to be adjusted and the element values should be modified (Feizizadeh and Blaschke 2013b). In our research, to use the advantage of spatial weighting of criteria, the pairwise comparison matrix was performed in Idrisi software. The derived weights are shown in Table 3 and Figure 3. Further, the resulting *CR* value was 0.053.

**Figure 3.**
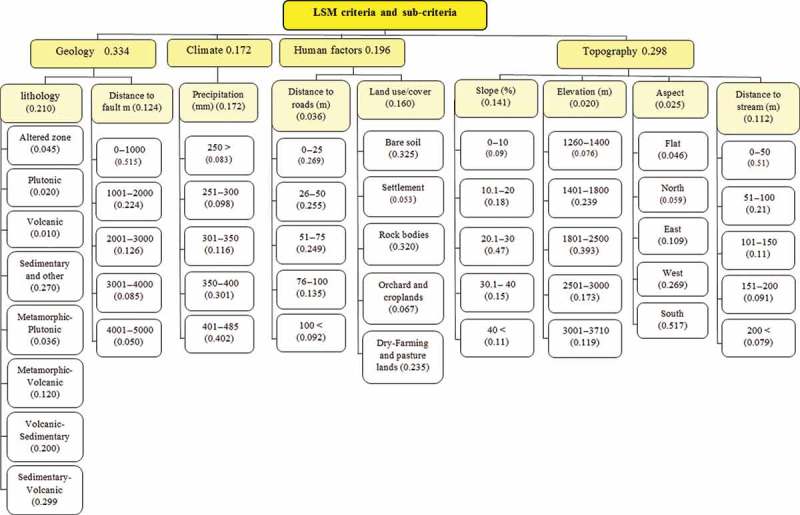
AHP decision tree for LSM.

**Table 3.** Pairwise comparison matrix for data set layers of landslide analysis.

Criterion	1	2	3	4	5	6	7	8	9	Weight
(1) Aspect	1									0.025
(2) Distance to road	1/5	1								0.036
(3) Elevation	1/2	1/3	1							0.020
(4) Distance to stream	1/3	1/3	1/3	1						0.112
(5) Distance to fault	1/3	1/5	1/5	1/3	1					0.124
(6) Slope	7	1/5	9	1/3	1/4	1				0.141
(7) Land use	8	6	1/5	1/5	1/3	1/3	1			0.160
(8) Precipitation	8	6	7	7	4	3	1/5	1		0.172
(9) lithology	9	7	1/3	8	7	4	1/5	8	1	0.210

Note: *CR*: 0.053.

### 3.4. Sensitivity in AHP’s pairwise matrix and sensitivity analysis in weightings of objectives and criteria

As discussed in Section 3.3, the AHP is a widely used method for criteria weighting in MCDA. However, since it considers the expert opinions for assigning weights, it essentially allows a degree of subjectivity in the pairwise comparisons between the criteria. As a result, any incorrect perception on the role of the different LSM criteria can easily be conveyed from the expert’s opinion into the weight assignment (Kritikos and Davies 2011, Feizizadeh and Blaschke 2013a). The uncertainty of weights lies in the subjective expert or stakeholder judgment of the relative importance of different attributes, given the range of their impacts (Chen *et al.* 2011). Therefore, after obtaining a ranking of alternatives, a sensitivity analysis may be performed to determine robustness. In the context of LSM, to reliably assess LSMs, sensitivity analyses show how a solution changes when the input factors are changed. If the selection of a factor results in a relatively large change in the outcome, the outcome is said to depend on that factor. Sensitivity analyses model behavior by determining the rate of change in the model output as parameters or by varying input data, thus giving an understanding of how changes in the inputs inﬂuence the output. Such analyses explain how inputs affect the output and quantify the strengths of the inputs based on the variations in the output (Oh *et al.* 2011). Figure 3 shows the AHP decision tree weights assessment for criteria and sub-criteria. The decision tree produced by the deterministic AHP approach for the MCDA model contains weights with discrete values. However, in practice, these values represent imprecise point estimates without indication of error or conﬁdence (Benke and Pelizaro 2010). The weights in the decision tree have a possible range of [0, 1]. If we arbitrarily set *x*_min_ = 0 for all weights at the lower bound and set *x*_max_ = 1 for all weights at the upper bound and then multiply all the weights through the hierarchy, in both cases (lower and upper bounds), the output range of the model would be the maximum possible. It would represent only the maximum theoretical range that values could assume for the speciﬁed ratings data. This result would have very limited use for uncertainty analysis (Benke and Pelizaro 2010).

### 3.5. Probability distributions

In the presence of epistemic uncertainty, classic probability theories are based on the representation of failure probabilities of components at a certain time using PDFs. The PDF *fi*(*x*) at time *t* indicates the probability that the value of the failure probability for a component *i* at time *t* falls between *x* and *x* *+ *d*x*. Probability theories based on MCS can then be used to evaluate the reliability of the whole system (Sallak *et al.* 2010). When moving from a deterministic approach to a probabilistic approach, these constraints must also be observed when sampling from the PDF used to represent uncertainty. The actual ratings at the base of the tree have associated uncertainties and are also sampled from a probability distribution. In this study, the PDF modeling variability of the weights was represented by the program evaluation and review technique (PERT) probability distribution. The PERT is a method to address uncertainty in the estimation of project parameters. The probability distribution of the individual weight parameters was assumed to be symmetrical, and the given minima and maxima were interpreted as the 99th percentiles of a normal distribution (Benke and Pelizaro 2010). The PERT distribution is distinguished by its smoothness and continuity, as well as its greater weighting to the most likely values rather than to the tails. Other advantages include computational ﬂexibility and greater suitability for modeling expert opinions because of its nonlinear nature. When the deterministic weight value in the decision tree is replaced by a probabilistic value, the sampling distribution of possible weight values is defined by the set {*x*_min_, *x*_mode_, *x*_max_}, where *x*_mode_ is the point estimate from the workshop experts, and the bounds are prescribed by the AHP (Benke and Pelizaro 2010). Constraint satisfaction requires nonnegative lower and upper bounds on weights and ratings, as defined by the AHP model inputs, and also the unit-sum normality condition on weights, *W_K_*, to hold for each layer in the AHP hierarchy, that is, for *K* = 1,2,...*m* criterion weights *X_k_*,

(3) 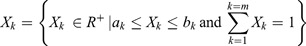

Note that *a_k_* and *b_k_* represent lower and upper methods, respectively, and together with *w_k_* are members of the set positive real members, *R* ^+^ The rating values at the base of decision tree are not subject to the normality condition. The PERT probability distribution is a special case of the *Beta* distribution. The probability density function for the *Beta* distribution is described succinctly by Croarkin and Tobias (2006) (see also Benke and Pelizaro 2010).

(4) 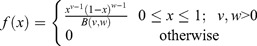

when the term in denominator is the *Beta* function,

(5) 
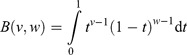

the shape parameters, *v* and *w*, are given by

(6) 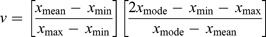

(7) 
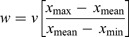

and the mean value, *x*_mean_, is defined as

(8) 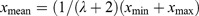

The scale parameter for the height of the distribution has the default value *λ* = 1. The PERT distribution is center-weighted but can be either symmetric or highly skewed, depending on the parameters. The ability to simulate a single peaked asymmetric distribution with predefined mode and range makes this a convenient model for representing uncertainty in the weights (as the original deterministic weights may be located near the boundaries of the feasible region) (Benke and Pelizaro 2010). Table 4 shows the minimum and maximum weights used for the PDF simulation. The AHP-based criteria weights were used as minimum weights along with user-defined maximum values for the simulation of PDF function, whereby the uncertainty analysis is intended to provide an indication of error bounds in the final result and take it into account of the LSM values by way of 1:1 correspondence. For this to happen, this process was pursued by performing the MCS as discussed in the following section.

**Table 4.** Minimum and maximum withed for PDF.

Factor	Reference weights	Maximum weights
Aspect	0.025	0.2
Distance to road	0.036	0.5
Elevation	0.020	0.55
Distance to stream	0.112	0.6
Distance to fault	0.124	0.7
Slope	0.141	0.75
Land use	0.160	0. 65
Precipitation	0.172	0.6
lithology	0.210	0.95

### 3.6. Simulation based on Monte Carlo method

A difficulty with traditional deterministic MCDA models is that the criterion weights in reality represent imprecise point estimates. The model prediction itself is also a point estimate with no indication of error or confidence (Benke *et al.* 2008, Benke and Pelizaro 2010). In an effort to deal with such sources of uncertainty and to overcome this deficiency, previous studies (e.g., Hus and Pan 2009, Benke and Pelizaro 2010, Feizizadeh and Blaschke 2013a, 2013b) have suggested to integrate MCS in conventional AHP in order to enhance the screening capability when there is a need to identify the best among the leading alternatives (Hus and Pan 2009). In our approach, we use the MCS to carry out the uncertainty analysis associated with AHP weights. For this to happen, our research methodology makes use of the concept of AHP-MCS, where we consider the criteria weights derived from the AHP pairwise matrix for the MCS uncertainty analysis. To implement AHP-MCS, the AHP decision tree was constructed for each criterion relevant to the LSM (See Figure 3). Uncertainties in the AHP decision tree occur in the weights, ratings, and landslide prediction (i.e., expert opinion, data, and predictions). The uncertainty associated with landslide predictions is determined by the sequential application of MCS in order to produce a series of model predictions for landslide susceptibility. Hereby, in each case, the corresponding uncertainty metric is computed from the simulation output PDF. Uncertainty in the weights was of paramount interest, and the extraction of marginal distributions was the prime objective (Benke and Pelizaro 2010).

The initial determination of point-estimates of weights depends on information available to the experts, in order that there is a component of ‘lack of knowledge’ or epistemic uncertainty, as well as ‘variability’ or aleatory uncertainty. As the uncertainty analysis uses a probability distribution to cover the full range of possible weight values, both cases are included in the MCS. Metrics for the quantification of aleatory uncertainty include standard deviation, variance, conﬁdence interval, and percentile data from the probability distribution (See applications by Benke and Hamilton 2008 and Benke *et al.* 2008). In recent studies, MCS often uses the software packages @RISK or the RANDOM function incorporated in the Idrisi software. Within our study, random samples were drawn from PERT for each weight parameter, which, based on qualitative information, had been defined via the mean value, as well as assumptions about the range of the relative importance of objectives. It is hypothesized that the public perception of the relative importance of management objectives will most likely always be heterogeneous (Elizondo *et al.* 2008).

**Figure 4.**
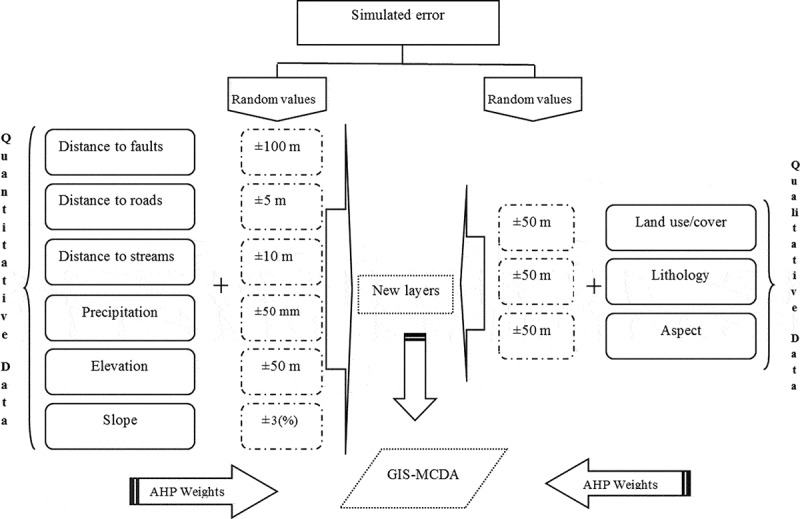
Scheme of MCSs to GIS-MCDA-based LSM.

In this study, we ran the MCS by considering *R* = 0.75 rating as the output of the LSM (mean and distribution) using @RISK. A series of MCSs were executed while incrementing the *R* value for each run (e.g., *R* = 0.50, 0.60, 0.75). Each run provided a LSM mean value, together with distribution data from @RISK. *R* = 0.75 was selected as the rating output of LSM, and simulations were run 100–10,000 times, depending on the computational load, the complexity of the model, and the desired accuracy. We obtained the distribution data from @RISK to address the spatial distribution of the diagnostic criteria. Accordingly, the RANDOM function in Idrisi software was used to add random values to the raster data set and to obtain new data sets for LSM (See Figure 4). It should be noted that the Idrisi RANDOM function allows the creation of the random data sets based on rectilinear, normal, or lognormal distribution models. In our study, the error of each factor was added to the deterministic part of the parameter using the normal distribution function in Idrisi. Normal distribution means that random values are generated with a likelihood following a normal distribution: values close to the mean are more probable and those far away will be less likely (Eastman 2006). For example, to add simulated error for distance to faults with an RMS error of ±100 m, a random function can be used to produce a layer using a normal model. Then, a simulation is performed on the original data using this random value for the particular layer such as distance to fault. For qualitative data (including lithology, land use/cover, and aspect), we introduced the simulated error by relocating the polygon borders. The result has no specific claim to reality, other than containing an error of the same nature as those believed to exist in the original. Figure 4 shows how the random replicates are used in the MCS.

### 3.7. Certainty analysis based on Dempster–Shafer theory

The DST of evidence, which was originally based on Dempster’s work on the generalization of the Bayesian theory (Dempster 1967) and was formalized by Shafer (1976), can provide a mathematical framework for the description of incomplete knowledge (Park 2011). DST is sometimes interpreted as an approximate generalization of Bayes’ rule but the priors and conditionals need not to be specified. One basic assumption of DST is that ignorance exists instead of supplying prior probabilities. It defines hypotheses in a hierarchical structure developed from a basic set of hypotheses that form the frame of discernment (Eastman 2012). DST provides additional flexibility for the specification of uncertainty in probabilistic models and hypothesis testing (Ducey 2001). It has mostly focused on uncertain reasoning in artificial intelligence and expert systems (Shafer and Pearl 1990). DST is suitable to reason with uncertainty and has been developed to overcome some limitations of conventional probability theory by distinguishing between uncertainty and imprecision. This is achieved in particular by making it possible to handle composite hypotheses (Kaftandjian *et al.* 2003). The DST of evidence is based on approximate reasoning where imprecision and uncertainties are introduced into the decision-making process by considering probability intervals with lower and upper bounds (Mertikas and Zervakis 2001, Gorsevski and Jankowski 2005). Dempster’s rule of combination provides a tool for combining multiple spatial data layers. The interval between belief and plausibility presents the uncertainty of the knowledge about the target proposition (Park 2011). In this theory, a frame of discernment *Θ* = {*T*_1_, *T*_2_,…, *T*_1n_}, which is a set of mutually exclusive and exhaustive propositions, is first established. Then, a mass function [*m*(*T*)] assigns the belief committed to each proposition, as shown (Park 2011):

(9) 
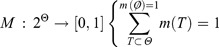

where 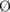 is the empty set.

**Figure 5.**
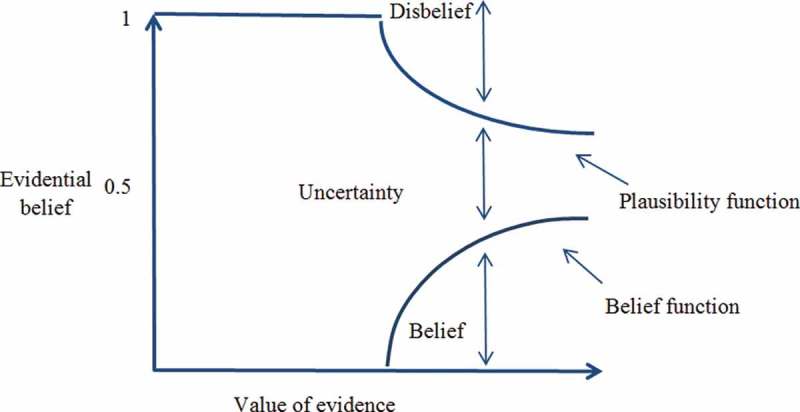
Schematic relationships of evidential belief functions (Althuwaynee *et al.* 2012).

Based on the mass function, the belief (Bel) and plausibility (Pls) functions are defined, respectively, by

(10) 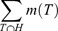

(11) 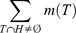

where for every 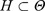, Bel(*H*) is a measure of the total amount of beliefs committed exactly to every subset of *H* by *m*. Pls(*H*) represents the degree to which the evidence remains plausible. These two functions, which are regarded as the lower and upper probabilities, respectively (An *et al.* 1994, Park 2011), have the following properties:

(12) 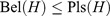

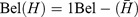

where 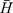 is the negation of *H* and Bel(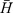) is also called the *disbelief function* (Park 2011). The above properties indicate that the unknown true probability or likelihood lies somewhere between the belief and plausibility functions. The difference between these two functions, also called the *belief interval* or ignorance, represents the ignorance of one’s belief of the target proposition *H* and can be regarded as a measure of uncertainty. This belief interval or ignorance is the main distinct characteristic of the DST of evidence compared to the traditional probability theory. Based on the DST approach, the belief function denotes the lower bound for an (unknown) probability function, whereas the plausibility function denotes the upper bound for an (unknown) probability function (See Figure 5 as a schematic representation of DST combinations). In this approach, the difference between the plausibility (Pls) and the belief (Bel) function represents a measure of uncertainty. The belief Bel and plausibility Pls functions for subset *A* are defined in the following manner:

(13) 
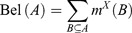

and

(14) 
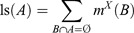

Bel(*A*) measures the total assignment of belief to *A* and all its subsets. The plausibility function measures the extent to which we assume the hypothesis of *A*. [Bel(*A*), Pls(*A*)] can be viewed as the confidence interval, which describes the uncertainty *A.* The functions Bel and Pls, although they too are functions that serve to map events of *A* onto [0,1] and Ø, 0, and *Ω* into 1, generally do not fulfill the sub-additivity properties for probability (Sallak *et al.* 2010). The ignorance value can be used to represent the lack of evidence (complete ignorance is represented by 0).

In our research, the DST approach was used to determine the certainty of LSM results. For this to happen, the belief function as implemented in the Idrisi software was used to carry out the spatial distribution of certainty for landslide susceptibility maps. The Belief function in Idrisi software aggregates the data from different lines of evidence by applying rules of combination based on Dempster–Shafer weight-of-evidence modeling. Each input data file contains basic probability assignments that indirectly relate evidence toward a hypothesis within a frame of decisions. The frame of decisions within a specific decision context includes all possible hypotheses and their hierarchical combinations. Belief constructs a knowledge base from the input data and the user-specified hypotheses each data layer supports. It then allows the user to extract *belief*, *plausibility*, and *belief interval* data layers for each hypothesis for which there is supporting evidence (Idrisi Selva 2012). To extract these three outcomes, our DST approach included the following steps (Eastman 2012):

- Creating a knowledge base file containing the hierarchical combinations of hypotheses in the decision set, a listing of evidence to aggregate, descriptions of each line of evidence, and the designations of the hypotheses for which evidence was created.
- Building a knowledge base by aggregating basic probability assignments from the evidence for all hypotheses, including those generated by the hierarchical combination of primary (singleton) hypotheses.
- Extracting summary images: a hypothesis for belief, plausibility, and belief interval images were extracted for the selected hypothesis from the constructed knowledge base.
- Aggregating new evidence to the existing state of knowledge and rebuild a knowledge base. The knowledge base is rebuilt if any lines of evidence are altered or if any of the knowledge base have been altered or deleted.

As a result, the belief, plausibility, and belief interval layers were derived. The belief layer provides a measure of the degree to which the evidence provides concrete support for the hypothesis. It is the lower bound of the belief in the hypothesis (Comber *et al.* 2010) and represents the degree to which the evidence provides concrete support for the proposition. It is important to interpret it together with the plausibility and belief interval images. Plausibility provides the upper boundary (Comber *et al.* 2010, Eastman 2012) and represents the degree to which the evidence does not refute the proposition. This actually represents the relationship of evidence to a particular outcome (hypothesis) (Eastman 2012). The belief interval records the range between belief and plausibility, providing a measure of the uncertainty in the hypothesis (Comber *et al.* 2010). The relationship between probability and belief interval layers is crucial for evaluating what decisions to make. Even if concrete evidence for a proposition is poor, that is, belief values are low, it is still possible to have high plausibility values in those areas. This would identify potential areas where enough information exists to make a concrete decision about the use of these spaces or the allocation of resources to them. At the very least, it will identify areas where the gathering of more evidence seems necessary. The uncertainty is the difference between plausibility and belief support and acts as a measure of uncertainty about a proposition (Tangestani and Moore 2002, Feizizadeh *et al.* 2012). The detailed results of DST for representing the certainty of LSM maps are discussed in Section 4.2.

## 4. Results

### 4.1. Producing initial landslide susceptibility maps

**Figure 6.**
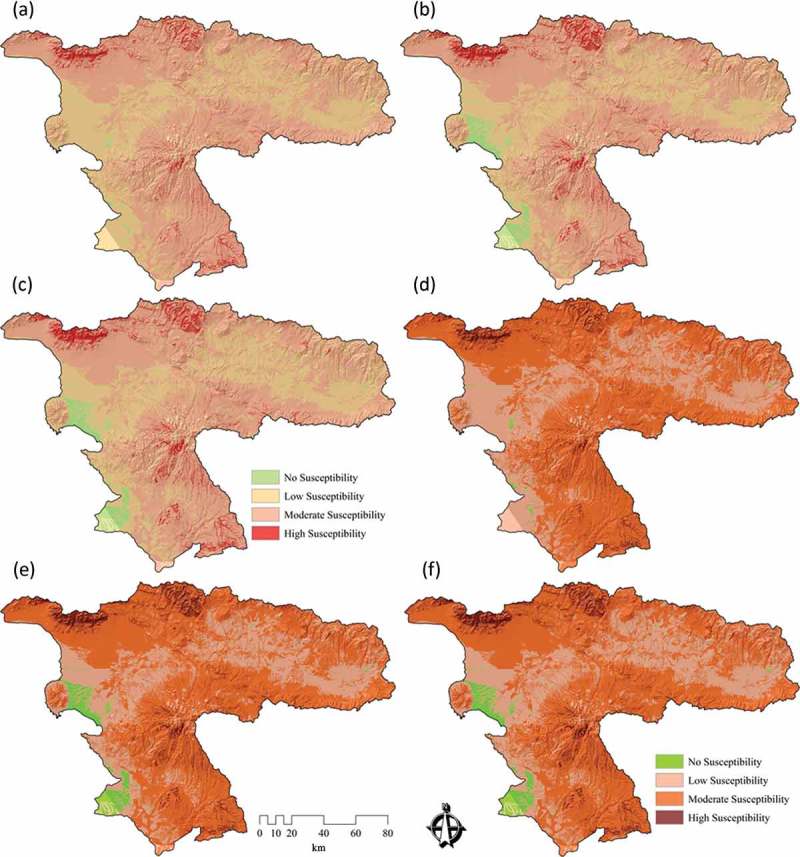
Landslide susceptibility maps (LSM) of ‘conventional’ and ‘novel’ approaches: (a) LSM derived from WLC, (b) LSM based on the AHP method, (c) LSM from the OWA method, (d) LSM derived from MCS-WLC, (e) LSM based on the MCS-AHP method, and (f) LSM from the MCS-OWA method.

The data sets were combined using the different MCDA methods, with a twofold analysis approach for the LSM. In the first approach, three landslide susceptibility maps were derived based on three different GIS-MCDA methods, namely, WLC, AHP, and OWA. Since this approach is based on a common GIS-MCDA methodology, it is called ‘conventional approach’. Figure 6a, b, and c depicts the landslide susceptibility maps derived by these three different methods of the ‘conventional approach’. Then, in a second step, we introduce a novel approach for GIS-MCDA by means of integrating MCS and GIS-MCDA under the concepts of sensitivity and uncertainty analyses. Here, susceptibility maps were developed based on the new raster layers, which were obtained from the MCS, resulting from the sensitivity and uncertainty analyses. Figure 6d, e, and f shows results of this novel LSM approach. To compare the different results and to assess the improved MCDA accuracies by means of sensitivity analysis, all six landslide susceptibility maps derived from both approaches were classified into four groups, namely, high, moderate, low, and no susceptibility to landslides, using the natural breaks classification method. The natural breaks method (or ‘Jenks optimization’) uses group values within a class, generating classes of similar values separated by breakpoints (Kritikos and Davies 2011, Feizizadeh and Blaschke 2013a).

### 4.2. DST for representing the certainty of results

**Figure 7.**
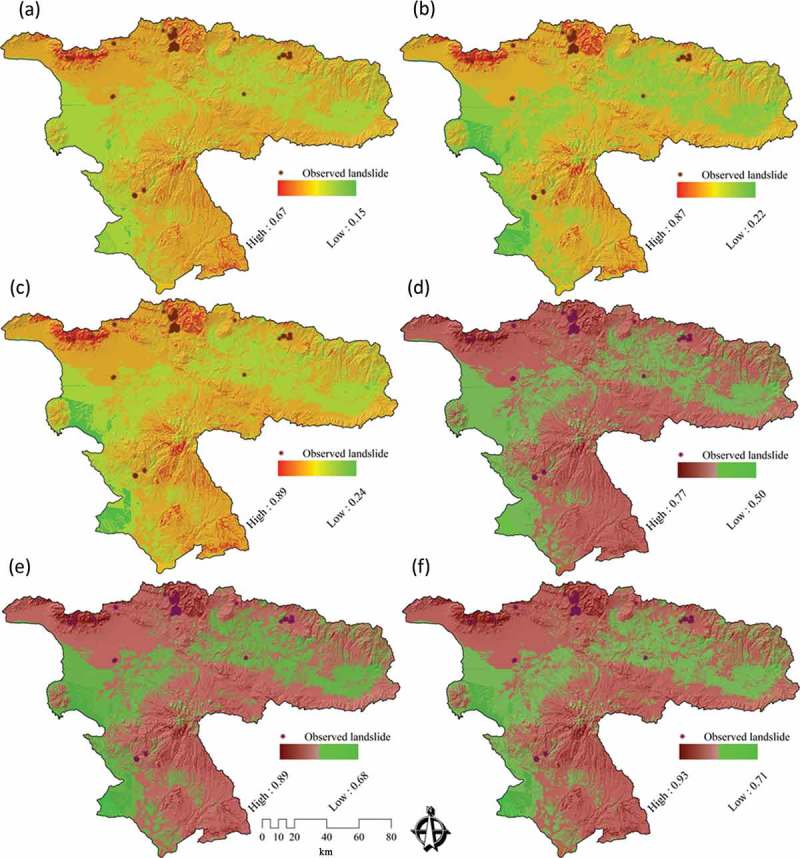
Uncertainty assessment (UA) for results of landslide susceptibility maps (LSM) of ‘conventional’ and ‘novel’ approaches: (a) UA results of the belief function for LSM derived from WLC, (b) UA results of the belief function for LSM based on the AHP method, (c) UA results of the belief function for LSM of the OWA method, (d) UA results of the belief function for LSM based on the MCS-WLC method, (e) UA results of the belief function for LSM of the MCS-AHP method, and (f) UA results of the belief function for LSM of the MCS-OWA method.

**Figure 8.**
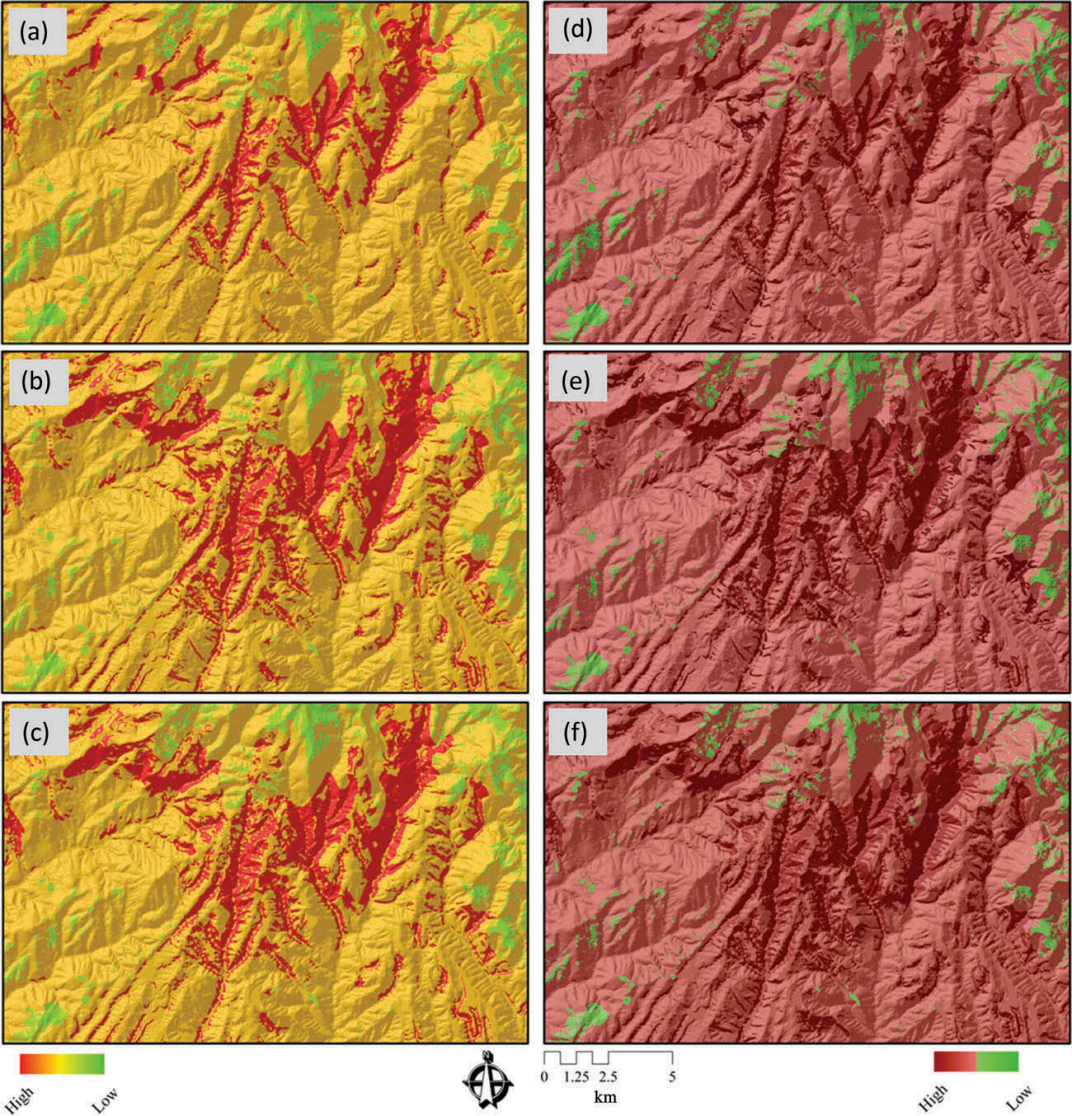
Results of UA for a selected area for ‘conventional’ and ‘novel’ approaches of landslide susceptibility maps (LSM) of (a) UA results of the belief function for LSM derived based on WLC, (b) UA results of the belief function for LSM based on AHP, (c) UA results of the belief function for LSM based on OWA, (d) UA results of the belief function for LSM based on MCS-WLC, (e) UA results of the belief function for LSM based on MCS-AHP, and (f) UA results of the belief function for LSM based on MCS-OWA.

As we discussed in Section 3.7, we used DST to visualize certainty of LSM results. Figure 7a, b, and c shows the certainty results of the conventional approach. Figure 7d, e, and f depicts the certainty of the maps derived from the novel approach. In the context of certainty assessment for the conventional approach of WLC method, the belief function reveals that certainty ranges between 0.15 and 0.67. This certainty range is significantly increased to 0.50–0.77 when the WLC method is employed in conjunction with MCS (MCS-WLC) within the novel approach. In terms of certainty assessment for the AHP method, the results show a certainty range of 0.22–0.87 for the conventional approach. However, this range is increased to 0.68–0.89 by integrating the AHP and MCS methods (MCS-AHP) in the novel approach. In the case of the certainty analysis for the OWA method, results of the conventional approach reveal a certainty range of 0.24–0.89, while the accuracy is significantly increased to 0.71–0.93 when integrating the MCS method (MCS-OWA) through the novel approach. To compare the spatial certainty and the differences between conventional and novel approaches, we present in Figure 8 an enlargement of a selected part of the study area. A cross-comparison of the certainty in GIS-MCDA results shows that the OWA method represents the greatest certainty range of all three MCDA methods used (see Figure 10a). The improvement indicates the flexibility of this method in association with other decision methods such as the MCS. This is important when trying to increase the accuracy of a MCDA. The detailed results of DST-based certainties for the different MCDA methods are listed in Table 5. The overall certainty and the improvement range for each method is also shown in Table 6 and Figure 10.

**Table 5.** Validation and certainty assessment (CA) of GIS-MCDA and GIS-MCS-MCDA results.

MCDA	Susceptibility category	Validation of MCDA results (%)	Validation of MCS-MCD results 2 (%)	CA of MCDA	CA of MCS-MCDA
WLC	High susceptibility	10.57	17.64	0.64–0.67	0.75–0.77
	Moderate susceptibility	86.33	79.26	0.56–0.63	0.71–0.75
	Low susceptibility	3.1	3.1	0.46–0.55	0.61–0.70
	No susceptibility	0	0	0.15–0.45	0.50–0. 60
	Sum	100	100	–	–
AHP	High susceptibility	21.2	29.41	0.85–0.87	0.86–0.89
	Moderate susceptibility	75.7	67.49	0.65–0.84	0.81–0.85
	Low susceptibility	3.1	3.1	0.46–0.64	0.76–0.80
	No susceptibility	0	0	0.22–0.45	0.68–0.75
	Sum	100	100	–	–
OWA	High susceptibility	20.16	29.26	0.85–0.89	0.86–0.93
	Moderate susceptibility	76.74	67.64	0.65–0.84	0.81–0.85
	Low susceptibility	3.1	3.1	0.46–0.64	0.76–0.80
	No susceptibility	0	0	0.24–0.45	0.71–0.75
	Sum	100	100	–	–

**Table 6.** Representation overall certainty and validation of results by DST and ROC curve.

Approach MCDA		Plausibility	Belief interval	Belief	AUC
Convectional	WLC	0.09–0.63	0.07–0.36	0.15–0.67	0.42451
	AHP	0.48–0.89	0.14–0.41	0.22–0.87	0.76912
	OWA	0.16–0.46	0.15–0.51	0.24–0.89	0.75645
Novel	MCS-WLC	0.21–0.71	0.23–0.57	0.50–0.77	0.71457
	MCS-AHP	0.52–0.91	0.44–0.63	0.68–0.89	0.87945
	MCS-OWA	0.63–0.92	0.49–0.71	0.71–0.93	0.83457

### 4.3. Validation of the models used

**Figure 9.**
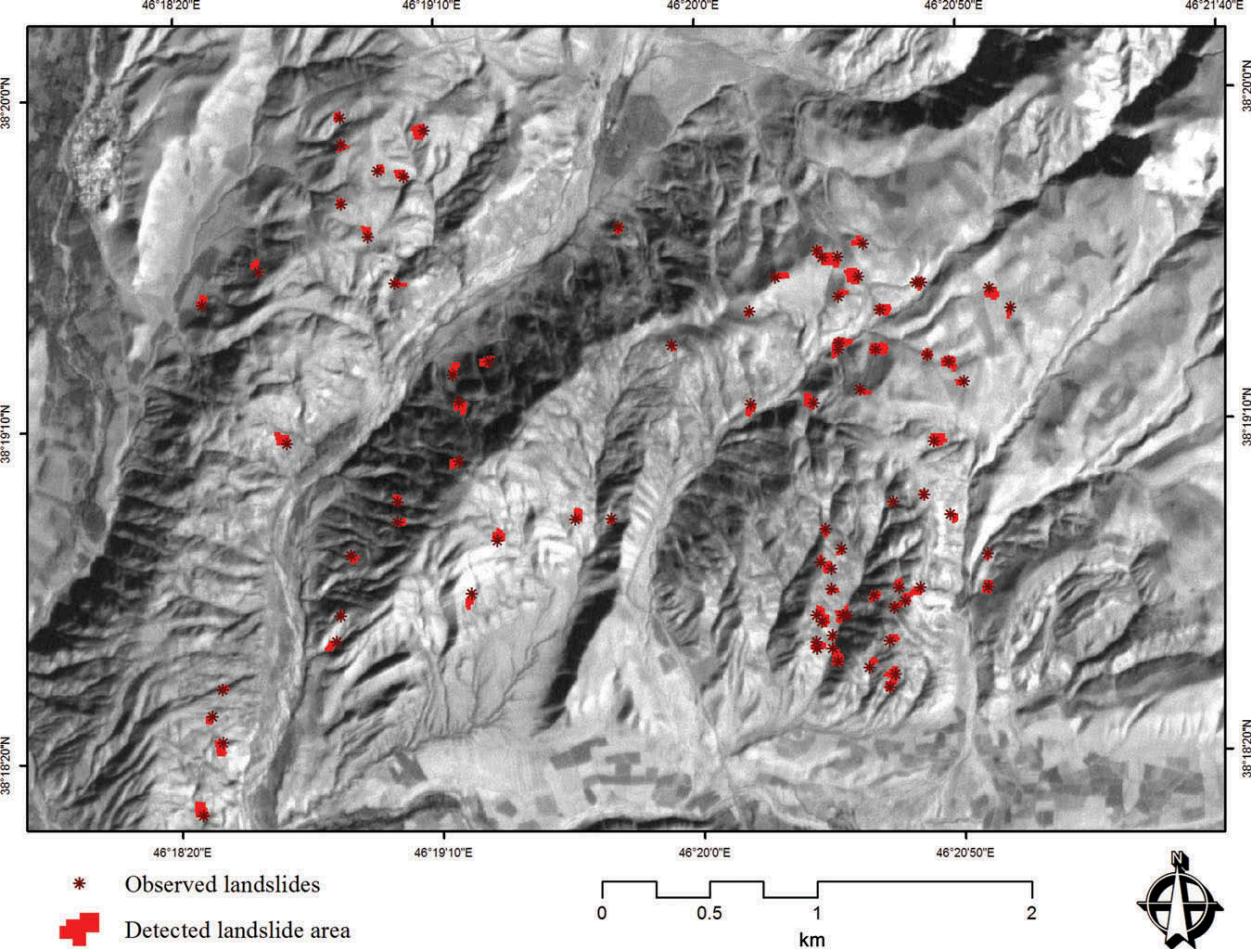
Landslide areas delineated by OBIA from IRS-ID satellite images (Feizizadeh and Blaschke 2013c).

**Figure 10.**
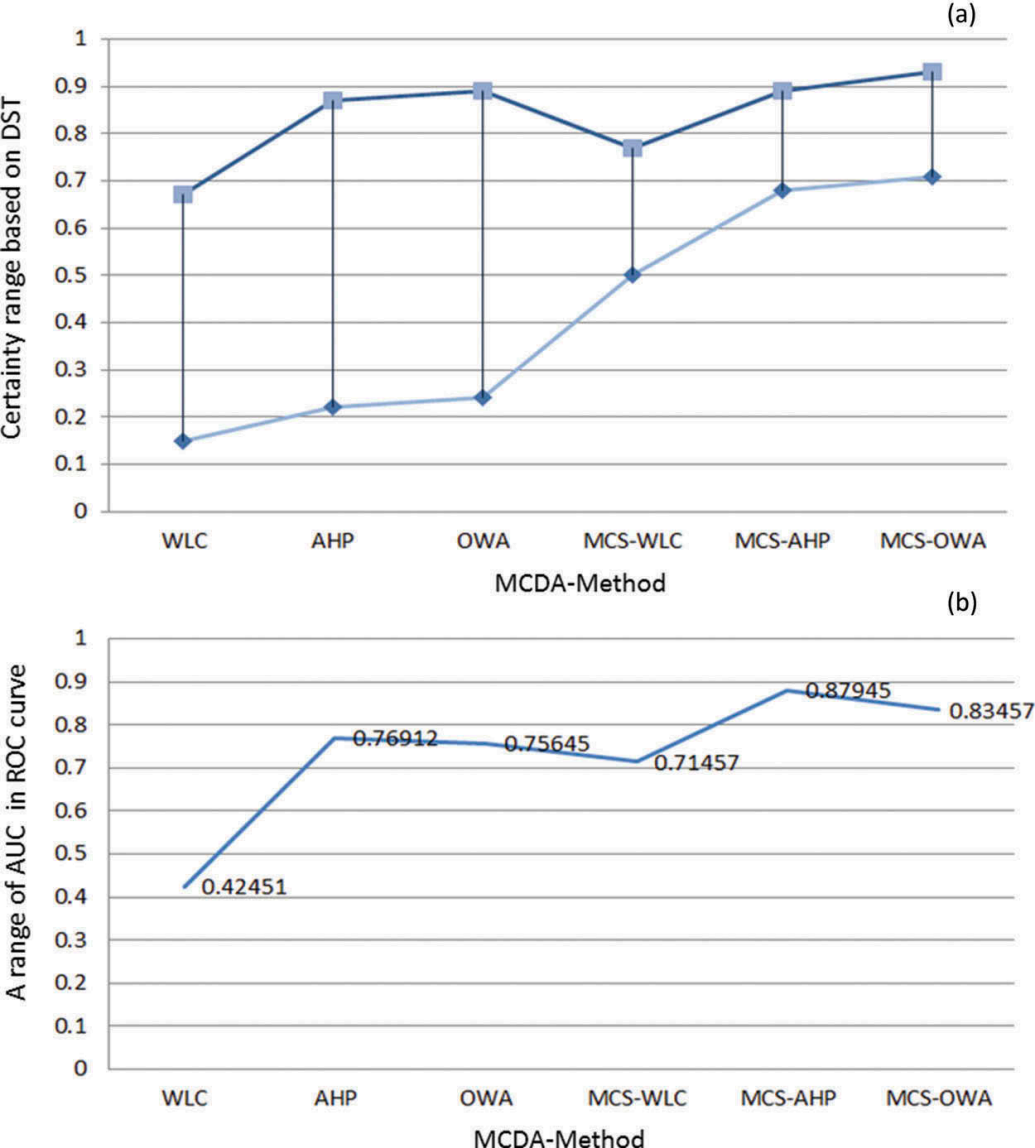
Comparative results of GIS-MCDA methods: (a) results of DST method for measuring the certainty and improved accuracy of GIS-MCDA by novel approach and (b) results of validation of methods based on ROC curve.

Validation is a fundamental part of the development of a susceptibility map, that is, for the determination of its prediction ability. The prediction capability of a landslide susceptibility model is usually estimated by using independent information such as a landslide inventory data set (Pourghasemi *et al.* 2012b). Within this research, to assess the accuracy of the LSM methods and to validate the results, the resulting LSMs were tested against known landslide locations within the study area. The landslide inventory database of the Urmia Lake Basin comprises 132 landslide events (MNR *et al.* 2010), but does not include exact outlines for these events. We therefore also use the results of a landslide delineation study based on IRS-ID satellite images (5.8 m spatial resolution). This study was previously carried out through object-based image analysis (Feizizadeh and Blaschke 2013c). Figure 9 shows the delineated landslide areas, which result in a total of 22.49 hectares. In doing so, the *r* *elative* *o* *perating* *c* *haracteristics* (ROC) (Nandi and Shakoor 2009) and the respective presences of landslide events (in percent) are calculated for each category for each landslide susceptibility map. The ROC curve is a plot of the probability of having a true positive (correctly predicted event response) versus the probability of a false positive (falsely predicted event response) as the cut-off probability varies (Gorsevski *et al.* 2006). This method has been widely used as a measure of performance of a predictive rule (Baeza *et al.* 2010). ROC plots the different accuracy values obtained against the whole range of possible threshold values of the functions, and the *a* *rea* *u* *nder the ROC* *c* *urve* (AUC) serves as a global accuracy statistic for the model, regardless of a specific discriminate threshold. This curve is obtained by plotting all combinations of sensitivities and proportions of false negatives (1-specificity), which may be obtained by varying the decision threshold. In the ROC curve, the ideal model shows a value close to1.0. However, the range of values of the AUC is 0.5–1 for a good-fit, while values below 0.5 represent a random fit (Pourghasemi *et al.* 2012b). The results of the ROC method for four landslide susceptibility maps are shown in Table 6 and Figure 10.

## 5. Discussion

Our research started off by applying sensitivity and uncertainty analyses for GIS-MCDA with the aim to improve the accuracy of the results and to compare three different GIS-MCDA methods to identify the most beneficial method amongst them. It is well know that the basic idea of any MCDA evaluation is primarily concerned with how to combine the information from several criteria to form a single index of evaluation (Gorsevski *et al.* 2012). In context of GIS-MCDA-based LSM, MCDA combines different layers of spatial information or factors to generate an aggregated measure of landslide susceptibility (Comber *et al.* 2010). In this regard, results of this research indicate that the integration of GIS-MCDA and MCS may improve the accuracy of GIS-MCDA significantly. Hereby, in the context of GIS-MCDA-based LSM, the AHP method performed best among three LSM methods, namely the AHP, WLC, and OWA. However, in terms of the certainty analysis, OWA represented the best reliability compared to the AHP and WLC methods. Finally, the WLC method delivered significantly poorer results for LSM, as well as for certainty analysis.

### 5.1. WLC method

It should be noted that the WLC is an aggregation method, which explains how different factors counterbalance each other, indicating their relative importance (Gorsevski *et al.* 2012). This method of GIS-MCDA is based on the concept of a weighted average in which continuous criteria are standardized to a common numeric range and then combined by means of a weighted average (Drobne and Lisec 2009). Technically speaking, WLC is often used for continuous factors in crisp MCDA processes (Voogd 1983). This approach uses compensatory aggregation rules where the decision set includes the overall value of the alternatives and where favorable criteria can outweigh unfavorable criteria (Gorsevski *et al.* 2012). The decision solutions obtained by the WLC method are located between the extreme ‘AND’ and ‘OR’ operators, while the decision sets are inﬂuenced by the weights representing the relative importance of the evaluation criteria. Weights given to each criterion determine the tradeoff level relative to other criteria, which implies that high scores and weights of some criteria can compensate for low scores and weights of other criteria. However, when scores from standardized criteria are low while the weights are high, they can only weakly compensate for the poor scores of other criteria (Jiang and Eastman 2000, Gorsevski and Jankowski 2010, Feizizadeh and Blaschke 2013a). Technically, the WLC approach suffers from three difficulties (Drobne and Lisec 2009). First, WLC used as a decision rule is influenced by the different aggregation methods employed in the decision-making process. Contrarily to an expectation that the WLC method and the Boolean method should yield similar results, they very often fail to do so because of logically different methods of aggregation. In the WLC method, a low score on one criterion can be compensated by a high score on another. This is different from Boolean options, which are absolute in nature. The second issue of the WLC stems from its standardization of factors. The most common approach here is to rescale the range to a common numerical basis by simple linear transformation. However, the rationale for doing so is unclear (Voogd 1983, Eastman *et al.* 1993, Drobne and Lisec 2009), and in some cases, a nonlinear scaling may seem appropriate. Third and most importantly, decision risk may be considered as the probability that the decision will fail. For a Boolean procedure, decision risk can be estimated by propagating measurement error through the decision rule, thereby determining the risk that the decision made for a given location can fail. However, in the context of continuous criteria of WLC, uncertainty is not so readily estimated with stochastic methods (Drobne and Lisec 2009). In an effort to deal with these issues, Jiang and Eastman (2000) suggested that those kinds of difficulties could be solved by considering decision-making as a set problem and through the application of fuzzy measures in multicriteria evaluation. They suggested that the OWA approach may provide an extension to and generalization of the conventional map combination methods in GIS (Drobne and Lisec 2009).

### 5.2. OWA method

The OWA method was originally developed in the context of fuzzy set theory (Jiang and Eastman 2000). Many decision-makers are happy with fuzzy representations of features, as the fuzzy method produces a more faithful picture, reflecting the continuum (Comber *et al.* 2010). OWA has been used in GIS context as method to overcome the systematic problems related to risk and tradeoff in MCDA (Comber *et al.* 2010). Using the OWA method, one can select any degree of tradeoff among the criteria ranging between no tradeoff and full tradeoff, according to the decision-making strategy (Jiang and Eastman 2000). OWA provides continuous fuzzy aggregation operations between fuzzy intersection (MIN or AND) and union (MAX or OR). It allows a variety of operators between MIN (AND) and MAX (OR), control over the degree of tradeoff between factors in MCDA and thereby allows the overall level of risk to be controlled (Comber *et al.* 2010). This is due to the fact that the OWA operators can fuse multi-attribute values into single aggregated values. It can be used to assign different weights to every attribute.

Based on the OWA approach, it is assumed that the decision-maker can intuitively identify the order weights based on the degree of ‘ORness’ (or ‘ANDness’) and tradeoff between criteria (Malczewski *et al.* 2003). To this end, it is important to notice that for a given value of ‘ORness’ one can obtain a large number of different sets of order weights and associated tradeoffs. Also, for a given degree of tradeoff, one can generate a large number of different sets of order weights and associated degrees of ‘ORness’ (Jiang and Eastman 2000, Malczewski *et al.* 2003). For a well-defined problem with clear and well understood parameters, OWA may offer obvious advantages: the factor and order weights can be used to constrain the aggregation process in a way that represents the current or best understanding of the problem being examined. However, this requires a very robust understanding of all the parameters involved in the decision and how they interact and influence the final outcome (Comber *et al.* 2010). In the context of landslide mapping, fuzzy methods, whether Min (AND), Max (OR), or in between, can provide a LSM full fuzzy model, which is able to better reflect the doubt in the minds of the decision-makers. In many situations where GIS is being applied to map suitability/susceptibility, decision-makers may not have a full understanding of how the application of MCDA methods (and therefore tradeoff and risk) relates to their problem and how weights interact with the resulting solutions. This informatics aspect is important when assessing tradeoff and risk in light of decision-making (Comber *et al.* 2010).

### 5.3. OWA/AHP combination

AHP has been suggested as a solution to the problem described in the last paragraph. AHP integrates fuzzy linguistic operators (Boroushaki and Malczewski 2008). However, the AHP still requires the domain knowledge and a clear definition of suitability/susceptibility to understand how to parameterize the input appropriately and then to interpret the rich and fuzzy output in light of those input decisions (Comber *et al.* 2010). Results of our research reveal that the OWA method, used in combination with criteria weights derived through the AHP approach, is very powerful for spatial decision-making. The integration of these two approaches has been verified to produce flexible and reliable results for LSM (Feizizadeh and Blaschke 2013a, 2013b). Results also demonstrate the possibility of improving accuracies of the methods by means of applying sensitivity and uncertainty analysis for MCDA methods. However, we should note that the differing accuracies are due to the different decision rules of the respective MCDA operators. Naturally, a variety of decision rules will result in various MCDA accuracies (Feizizadeh and Blascke 2013a). Nevertheless, based on the results of this study, we can conclude that the variety of options in MCDA may almost always yield variable results, and therefore, uncertainty analysis can be considered as a solution for the reliability assessment, as well as for the selection of the most appropriate method.

## 6. Conclusions and future work

The purpose of this work was to illustrate the resulting variabilities for different MCDA methods in the light of decision-making with and without sensitivity and uncertainty analysis approaches. We carried out a GIS-MCDA uncertainty analysis and demonstrated a solution for the uncertainty modeling by introducing a new approach for GIS-MCDA. In conclusion, the work has explored different methods for combining spatial data in a multi-criteria evaluation of landslide mapping. Based on the results, we can conclude that the parameterization of fuzzy MCDA approaches (e.g., OWA) requires a full understanding of how the factors’ tradeoffs against each other determine the resulting uncertainty. Results also indicated that different MCDA methods can modify the original factor weights and produce either very conservative or very liberal results in between (tradeoff) mappings of landslides (Comber *et al.* 2010). The novel approach described could significantly improve the results of GIS-MCDA.

The authors hypothesize that it should become obligatory to assess the reliability of the methods used for GIS-MCDA. This work has also shown that in situations where expert opinions for whatever reason are not available for the parameterization of the MCDA operation (in terms of factor weights, order weights, degrees of acceptable tradeoff, and thresholds to interpret the resulting aggregation), the DST can provide an alternative or complement to ‘traditional’ fuzzy MCDA approaches for suitability analyses. In such situations, the outputs of DST-MCDA may be more appropriate than a fully fuzzy model of suitability (Comber *et al.* 2010).

Future research is foreseen, which will include the application of the DST and spatially explicit reliability models for spatial sensitivity and uncertainty analyses of GIS-MCDA. The integration of a fuzzy set with GIS-MCDA (Fuzzy-AHP and OWA) and certainty analysis of the results will also be addressed in future work in order to make use of the flexibility of the OWA method for LSM. Our future work will include neural networks and comparisons with frequency ratio and bivariate logistic regression modeling for LSM. We plan to study the accuracy of these approaches and will assess them through certainty analyses using DST and fuzzy-AHP MCDA. In this regard, we may emphasize the importance of accuracy in landslide susceptibility maps, when it is used as a basis for decision-making plans in order to reduce and mitigate further landslide hazards. We conclude that the six resulting landslide prediction maps were not only accomplished for the sake of comparison. We will provide all six versions with respective explanations to the responsible authorities in the East Azerbaijan province for risk management. The information provided by these maps shall help citizens, planners, and engineers to reduce losses caused by existing and future landslides by means of prevention, mitigation, and avoidance.
